# Is glucagon action altered in steatotic liver disease?

**DOI:** 10.1172/JCI202425

**Published:** 2026-03-02

**Authors:** Raghavendra G. Mirmira

Metabolic dysfunction–associated steatotic liver disease (MASLD) commonly accompanies obesity and increases the risk for type 2 diabetes. In this context, hepatic insulin resistance promotes inappropriate glycogenolysis and gluconeogenesis, contributing to fasting and postprandial hyperglycemia. Growing interest in the pharmacologic potential of glucagon and related peptides has drawn new attention to the physiologic roles of glucagon signaling in obesity and MASLD. Hyperglucagonemia is now recognized to further amplify hepatic glucose production, compounding insulin resistance and exacerbating hyperglycemia (1). Whether hyperglucagonemia results from dysfunction of the α cell or dysregulation of a liver-to–α cell feedback axis in humans has been debated (2, 3). In this issue of the *JCI*, Christie et al. (4) utilized human insulin clamp studies and splanchnic gradient analyses, finding no evidence for dysregulation in the liver-to–α cell axis and suggesting that α cell dysfunction itself may be the predominant cause of hyperglucagonemia.

The physiologic actions of glucagon on the liver can be delineated into two molecular arms: the “carbon” arm and the “nitrogen” arm. The carbon arm reflects the actions of glucagon to stimulate glycogenolysis and gluconeogenesis to collectively promote hepatic glucose production. By contrast, the nitrogen arm of glucagon action increases hepatic amino acid uptake/clearance, drives amino acid deamination, and is coupled to urea cycle activation and urea synthesis, thereby disposing of amino-nitrogen (5). Because amino acids strongly stimulate glucagon secretion, the nitrogen arm functions as a negative feedback loop whereby hepatic amino acid uptake and metabolism diminish the signals that promote ongoing glucagon release by α cells. Based on this understanding of glucagon physiology, earlier work hinted that MASLD leads to selective glucagon resistance in the liver with an intact carbon arm but impaired nitrogen arm (3, 6). Resistance in the nitrogen arm would consequently account for the persistence of hyperglucagonemia in MASLD.

To test for selective glucagon resistance, Christie et al. (4) performed elegant human physiology studies combining arteriovenous catheterization to assess splanchnic amino acid balance and leucine tracer–based measurement of amino acid metabolism, together with clamp experiments designed to approximate a postprandial hormonal milieu and deliver graded doses of glucagon. These studies were conducted in lean people as well as obese people with or without hepatic steatosis (defined by a hepatic proton density fat fraction ≥ 5% by MRI). Across these groups, glucagon-induced changes in leucine metabolism and splanchnic amino acid balance were remarkably similar, indicating that hepatic steatosis does not impair acute glucagon-mediated regulation of amino acid metabolism. These findings are consistent with a recent prior report (2) but extend the field by directly measuring hepatic amino acid flux and tracer-based metabolism, thereby providing a more conclusive assessment of hepatic glucagon action.

While Christie et al. (4) address key methodological limitations that have confounded prior interpretations of hepatic glucagon resistance, their findings also shift emphasis toward the α cell, raising the possibility that α cell–intrinsic defects in nutrient sensing or intraislet communication underlie hyperglucagonemia in MASLD. Future studies that directly interrogate α cell physiology in humans will be critical to determine whether dysregulated glucagon secretion, rather than impaired hepatic action, represents the primary driver of this disorder.

## Funding support

This work is the result of NIH funding, in whole or in part, and is subject to the NIH Public Access Policy. Through acceptance of this federal funding, the NIH has been given a right to make the work publicly available in PubMed Central.

- NIH R01 DK060581.

## Version 1 03/02/2026

Electronic publication
